# Management of Severe Bilateral Axillary Hidradenitis Suppurativa in a Resource‐Limited Setting: A Case Report and Review of the Literature

**DOI:** 10.1002/ccr3.71176

**Published:** 2025-10-06

**Authors:** Nelson Mosha, Alex Mremi, Jere Mshana, Lulyritha C. Kini, Daudi Mavura, Gregor Jemec

**Affiliations:** ^1^ Regional Dermatology Training Centre Moshi Tanzania; ^2^ Kilimanjaro Christian Medical Centre Moshi Tanzania; ^3^ School of Medicine KCMC University Moshi Tanzania; ^4^ Kilimanjaro Clinical Research Institute Moshi Tanzania; ^5^ Zealand University Hospital, University of Copenhagen Copenhagen Denmark

**Keywords:** axilla, bilateral, hidradenitis suppurativa, limited resources, management challenges, surgery

## Abstract

In severe hidradenitis suppurativa, early recognition and prompt surgical management offer effective and lasting disease control, especially where advanced medical therapy is unavailable. Even advanced cases may be successfully treated with relatively simple procedures such as wide local excision, which is associated with favorable outcomes and low recurrence rates.

## Graphical abstract

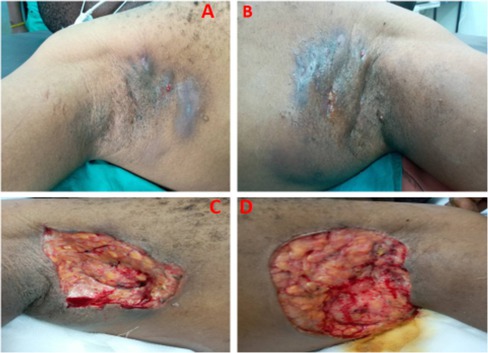

## 1 Introduction

Hidradenitis suppurativa (HS) is a chronic, inflammatory skin condition which, by definition, consists of specific morphology, periodic flare 2+/6 months, and affects apocrine gland–bearing areas such as the axillae, groin, and perineum [1]. It is characterized by recurrent painful nodules, abscesses, and, in contrast to most inflammatory skin diseases, HS results in tunnel formation and scars [2]. It is estimated that HS prevalence in Europe, the United States, and Australia ranges between 0.00033% and 0.4% [3]. Women are more affected compared to men [1]. The disease condition is the highest among patients aged 30–39 years, and in US studies appears more than three times as common in African American patients than Caucasian patients [2].

The etiology and pathogenesis of HS are poorly understood. However, several factors, including smoking, obesity, insulin dysregulation, skin microbiota imbalance, and environmental influences, contribute to the chronic inflammation characteristic of HS [4]. Heat, sweating, physical activity, shaving, and friction may exacerbate symptoms. Similarly, numerous associations, including obesity, smoking, hypertension, thyroid disease, dyslipidemia, and psychiatric disease, have been implicated [5]. Usually, alternating episodes of exacerbations and quiescence are typical features [2].

Clinically, the characteristic HS lesions are deep‐seated, painful, recurrent nodules that expand to form abscesses, which may subsequently rupture and drain, and eventually form scars [1]. These symptoms significantly impair patients' physical, social, and functional well‐being, contributing to a profound negative impact on quality of life. Patients often endure recurrent flares associated with pain, malodor, and chronic disfiguring lesions, compounding the disease burden [2]. Advanced stages may lead to disability and psychosocial distress [6]. An increased risk of major adverse cardiovascular events, including myocardial infarction and stroke, as well as all‐cause mortality, has been documented [6].

The treatment of HS remains a formidable challenge due to the absence of a definitive cure. The current management strategies aim to reduce symptoms, control inflammation, and prevent recurrence. Options include topical and systemic antibiotics, and surgical interventions for advanced disease [7]. Despite these measures, recurrence rates remain high. Biologic agents, such as adalimumab, secukinumab, and bimekisumab, have demonstrated significant efficacy in reducing disease recurrence and severity [8]. However, access to biologics is limited in resource‐constrained settings, including our institution. The universal requirement for any physician is to take into account not only potential advantages for the patient but also health, economic, and other host risks.

Herein, the authors describe a case of a patient with bilateral axillary HS that was successfully surgically treated after 2 years of unsuccessful medical treatment and a brief review of the pertinent current literature. The case highlights the importance of surgical management and the adaptability required in resource‐constrained environments to achieve favorable outcomes.

## 2 Case Examination

**FIGURE 1:**
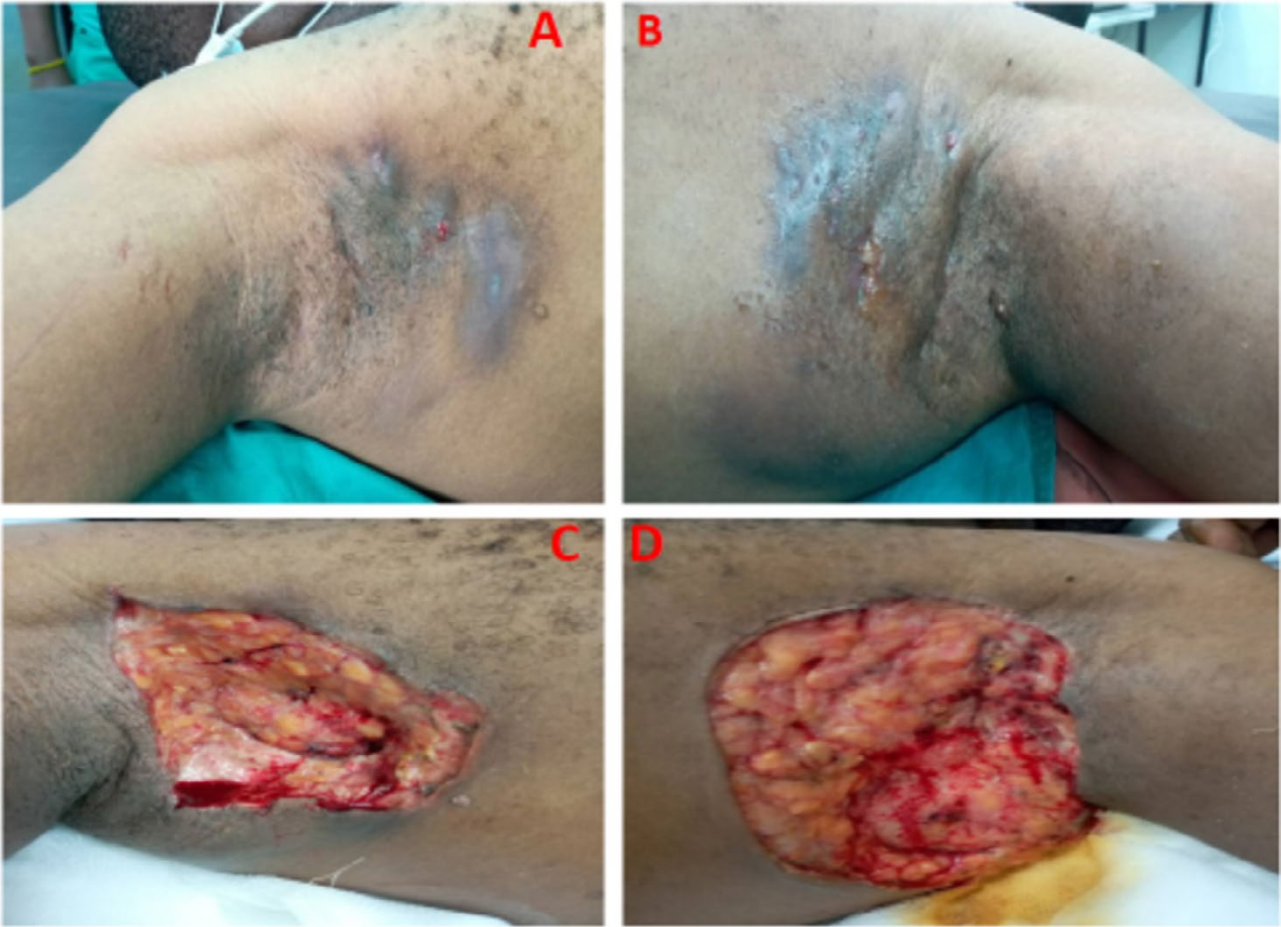
Clinical presentation of hidradenitis suppurativa lesions showing nodules and discharging fistula with scarring on the right axilla (A), and left axilla (B); photographs of the patient after a radical excision surgery of the entire affected regions (C, D).

A 37‐year‐old male presented with a 2‐year history of multiple recurrent, bilateral discharging axillary sinuses associated with foul‐smelling, purulent, and blood‐stained discharge. The condition progressively worsened and significantly impaired the patient's quality of life. On general examination, the patient was morbidly obese with a BMI of 35 kg/m^2^. Both axillae revealed multiple discharging sinuses, extensive scarring, and granulating tissue (Figure 1A,B). There was tenderness and malodor from the affected areas. His past medical history revealed a history of type 2 diabetes mellitus, and he was seropositive for hepatitis B surface antigen (HBsAg). The patient reported a history of receiving medical treatment for his condition that included topical and systemic antibiotics as well as intralesional corticosteroids. However, no improvement was appreciated.

## 3 Methods

His laboratory investigation results highlighted elevated total cholesterol and serum triglycerides (2.47 mmol/L), while the liver function test showed a slight elevation in aspartate transaminase (AST) and alanine transaminase (ALT). The leading clinical diagnosis of Hurley Stage III HS was considered, with differential diagnoses of ruptured epidermal inclusion cyst and folliculitis. The patient was discussed in a specialist multidisciplinary meeting, and sustained intensifization traditional surgery was recommended after the patient's diabetes and liver issues were kept under control.

## 4 Surgical Technique

A wide local excision was performed in the theater under general anesthesia with supplemental local anesthesia (1% lidocaine with 1:100,000 epinephrine) infiltrated in the skin surrounding the diseased area. The tunnels were injected with methylene blue. Complete excision of all involved skin and subcutaneous tissue was performed (Figure 1C,D). The depth of dissection was guided by the extent of disease, frequently heralded by the presence of methylene blue. The depth varied from the level of Scarpa's fascia to the muscular fascia. The wounds were dressed with topical antimicrobial medication guided by wound culture results and a sterile dressing.

## 5 Postoperative Wound Care

The aim of postoperative wound care was to maintain an optimal moist and clean wound, which was achieved with regular wound dressing changes, hydrotherapy, and physical therapy. Topical antimicrobial dressing changes to the affected areas were initiated on postoperative day one and continued until complete wound healing was achieved. The patient underwent hydrotherapy and physical therapy evaluation and treatment on postoperative day one, which was continued as needed on an outpatient basis in order to maintain an appropriate range of motion (ROM) and prevent scar contracture. The patient was followed up 1 week postoperatively and subsequently every few months until complete wound healing was achieved.

**FIGURE 2:**
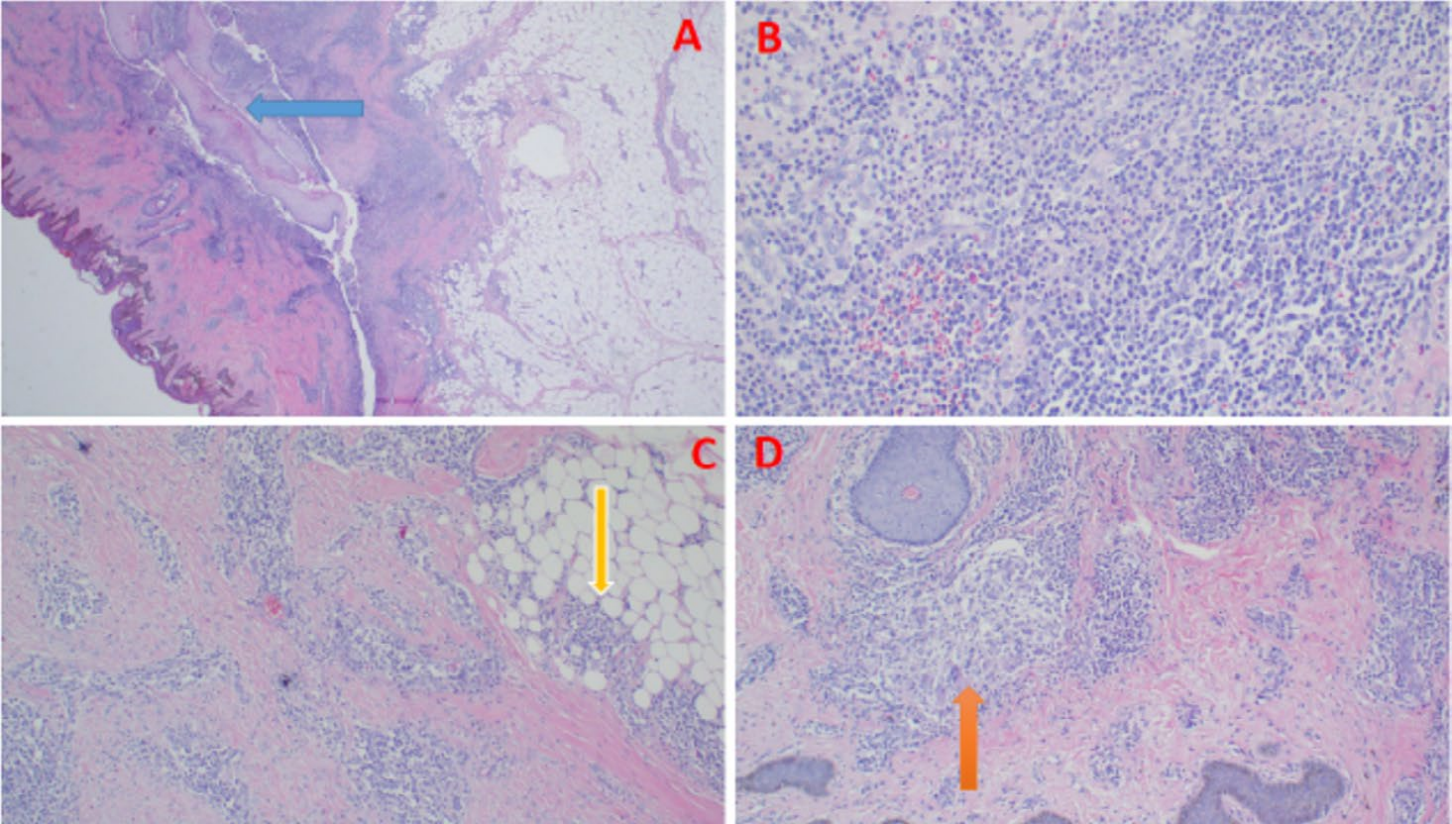
Histopathology of excisional biopsy of the hidradenitis suppurativa lesion demonstrating a ruptured dermal sinus tract (blue arrow) surrounded by mixed inflammatory infiltrates and neutrophils (A), photomicroscopy of the lesion highlighting neutrophilic abscess (B), an extension of the inflammatory cells into the subcutis (yellow arrow), (C); granulation tissue with occasional granuloma with foreign body giant cells (orange arrow) (D).

The obtained surgical specimen was sent for histopathology evaluation. The histopathology report described a chronic inflammatory skin disease of the pilosebaceous apocrine unit characterized by nodules, abscesses, fistulae, and sinus tracts, with scarring. The morphological features were consistent with HS (Figure 2A–D). The patient was kept on topical and systemic antibiotics for better disease control.

## 6 Conclusion and Results

**FIGURE 3:**
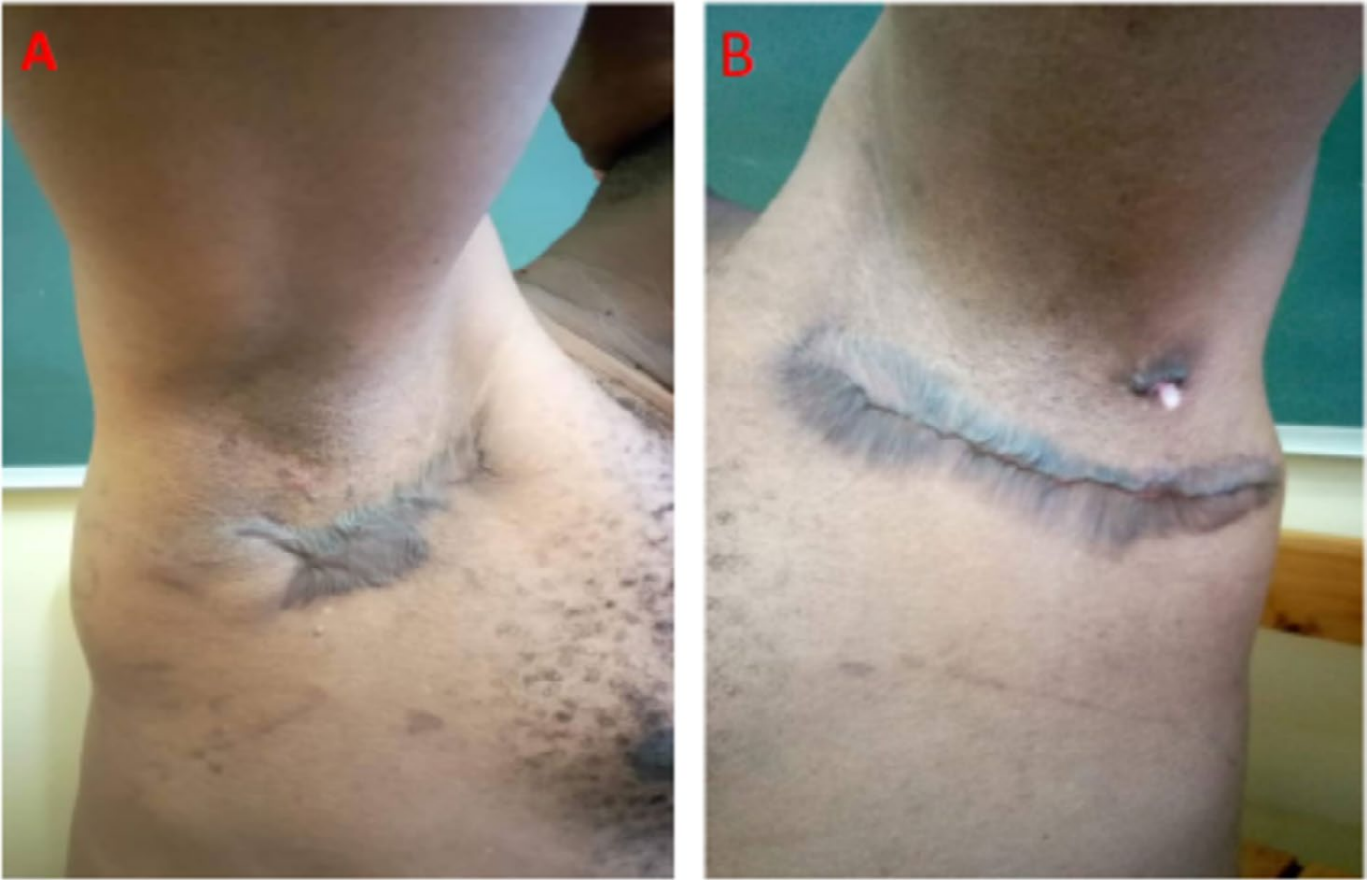
Appearance of the patient 4 weeks after local wide excisional surgery demonstrating a secondary intention healing of the right axilla (A) and left axilla (B), respectively.

Postoperative recovery was uneventful during the subsequent follow‐up visits (Figure 3A,B). To date, at least 12 months have passed since the patient completed treatment, and the patient remains disease‐free without signs of recurrence.

## 7 Discussion

The present case highlights that it is possible to manage recurrent HS lesions by repetitive wide surgical removal of the lesions and subsequent healing by second intention [9]. The aggressive physical removal of diseased tissue offers a practical solution even in a low‐resource setting and should therefore be kept in mind even though the literature abounds with more costly options.

HS is a complex disease that involves inflammation as well as scarring and tunneling, that is, transient as well as permanent tissue damage. Early recognition and treatment are therefore of importance. Despite this diagnosis, it is frequently delayed up to several years, as was evidenced in our patient. The diagnosis is made from clinical observation and the disease narrative [10]. Three criteria are being used, namely characteristic lesions, obligatory involvement of one or more flexural sites, and lesion recurrence (> 2×/6 months) [4]. Phenotypic variation renders diagnosis and severity assessment difficult. Ultrasound imaging is an emerging assessment tool for deep‐seated lesions. Ultrasound may aid in the assessment of disease severity [10]. Typically, the demonstration of thickened dermis, widened hair follicles, tracts, hypoechoic fluid pockets, decreased surrounding tissue echogenicity, and abscesses are characteristic features of HS. The Hurley's 3‐tiered staging system is used to describe disease severity and help select treatment [11]. Recent systems such as Sartorius, HSSI, and HiSCR have been designed to quantify disease intensity and response to treatment [12].

Managing HS remains a significant challenge, especially in resource‐limited settings. The treatment of HS is particularly challenging due to limited access to advanced therapies [13]. Core treatment modalities often include topical, intralesional corticosteroids, and systemic therapies such as clindamycin and rifampin antibiotics, along with surgical interventions like deroofing or wide local excision [8, 12, 13]. The treatment requires a multidisciplinary approach involving dermatologists, surgeons, and other healthcare professionals. Other treatment strategies include biologic agents targeting pro‐inflammatory cytokines (e.g., tumor necrosis factor‐alpha and interleukin‐17), offering promising results in moderate to severe cases refractory to conventional treatments [14]; but to the best of our knowledge, no direct comparisons have been made.

We suggest that the management of HS optimizes the use of available resources, including antibiotics and surgical interventions. Early surgical intervention has shown promising outcomes, but recurrence remains a major concern. Our patient previously received medical treatment, but no improvement was observed initially. This led us to pursue a more aggressive surgical approach. Multimodal therapy does not mean that the different treatments are weighted equally in all cases.

Our case study highlights the critical role of surgery in managing HS in resource‐limited settings. It shows that wide surgical excision as monotherapy may provide a long‐lasting, possibly definitive resolution of the disease, including removal of scar tissue [15, 16]. Only the simplest techniques were used: a wide local excision followed by secondary healing.

In addition to practical technical considerations directly associated with surgery, we were also aided in the decision by the patient's preference.

The presence of chronic hepatitis B infection, as indicated by positive HBsAg, adds a layer of complexity to the management. Liver dysfunction in this patient may influence the pharmacokinetics of medications and restrict therapeutic options. The elevated liver enzymes (AST and ALT) observed warrant close monitoring to mitigate the risk of drug‐induced hepatotoxicity, particularly when systemic therapies are being considered. All this complexity favored the surgical intervention in this patient. For routine care, maintaining local hygiene and selecting appropriate dressings considering lesion morphology are key considerations. Routine wound care should be adjusted to individual patient preferences as well. For postsurgical care, management of infection and nonviable tissue should be evaluated throughout healing. Different reconstructive techniques will influence wound care recommendations [17]. Preoperatively, medical optimization and discussion of wound healing expectations should be executed. Routine and postsurgical wound care for patients with HS have a high clinical, financial, and humanistic burden [18].

Multimodal therapy remains a cornerstone in the comprehensive management of HS, with each component—medical, surgical, and supportive care—playing an integral role [7, 19]. While the availability of medications is important, effective management does not necessarily depend on high‐cost biologic therapies. In many cases, other readily available medications can provide significant relief. Moreover, surgical intervention combined with general supportive care can still yield meaningful benefits for patients when medications are unavailable, inaccessible, or contraindicated [20]. This underscores the importance of tailoring treatment strategies to the resources available, ensuring that patients in resource‐limited settings receive the best possible care. The adaptability and effectiveness of multimodal treatment approaches are particularly valuable in addressing the challenges posed by HS in low‐resource environments. By leveraging low‐cost surgical techniques and emphasizing the importance of supportive care, healthcare providers can make a significant impact on the lives of patients suffering from this debilitating condition [19, 20].

While the case report offers valuable insights into the clinical management of severe bilateral axillary HS in a resource‐limited setting, several limitations must be acknowledged. As a single‐patient case study, the findings lack generalizability, particularly given the heterogeneous nature of HS across different populations and disease sites. The absence of a control or comparison group restricts the ability to evaluate the efficacy of the intervention relative to standard treatments, particularly biologic therapies that may be inaccessible in the described setting. Furthermore, the diagnostic and therapeutic decisions in the index case were influenced by resource constraints, which may limit the applicability of the reported approach to settings with broader healthcare infrastructure. The report also suffers from potential bias, as successful cases are more likely to be published, and the authors' conclusions may not be supported by objective, long‐term outcome measures. In addition, the accompanying literature review may not comprehensively address recent advances in HS management or critically appraise the evidence presented. Lastly, the lack of extended follow‐up raises concerns about recurrence and the sustainability of the clinical outcome. Despite these limitations, the case report remains a useful contribution, highlighting the challenges and adaptations required in managing chronic dermatological conditions within under‐resourced health systems.

## 8 Conclusion

Severe bilateral recurrent axillary hidradenitis suppurativa can be effectively managed in resource‐limited settings using meticulous clinical assessment, surgical excision, and appropriate postoperative care. While biologics and advanced wound care modalities may not be available, fundamental principles of surgical management remain accessible and impactful. This case highlights the importance of adapting care strategies to local contexts and calls for increased awareness and training in HS management in underserved regions.

## Author Contributions

**Nelson Mosha:** conceptualization, data curation, methodology, writing – original draft. **Alex Mremi:** conceptualization, data curation, investigation, methodology, writing – review and editing. **Jere Mshana:** data curation, methodology, writing – review and editing. **Lulyritha C. Kini:** data curation, methodology, writing – review and editing. **Daudi Mavura:** conceptualization, data curation, methodology, writing – review and editing. **Gregor Jemec:** conceptualization, data curation, methodology, writing – review and editing.

## Ethics Statement

The patient provided written informed consent to allow his de‐identified medical information to be used in this publication. A waiver for ethical approval was obtained from the authors' institution review board (IRB) committee since case reports do not need IRB approval.

## Consent

Written informed consent for publication of clinical details and images was obtained from the patient.

## Conflicts of Interest

The authors declare no conflicts of interest.

## Data Availability

The authors have nothing to report.
